# Mid-Term Outcomes of Novel Covered Stent with Biodegradable Membrane in Porcine Coronary Artery Perforation

**DOI:** 10.31083/j.rcm2407197

**Published:** 2023-07-12

**Authors:** Wei Cai, En Chen, Hong Zheng, Danqing Hu, Lingzhen Wu, Xiaoling Zeng, Jinhua Huang, Lianglong Chen

**Affiliations:** ^1^Department of Cardiology, Fujian Heart Medical Center, Fujian Institute of Coronary Artery Disease, Fujian Institute of Geriatrics, Fujian Medical University Union Hospital, 350001 Fuzhou, Fujian, China; ^2^School of Health, Fujian Medical University, 350005 Fuzhou, Fujian, China

**Keywords:** coronary artery perforation, covered stent, poly-L-lactic acid, biodegradable membrane, porcine, fibroplasia, neoatherosclerosis

## Abstract

**Background:**

Currently, commercially covered stents are the main treatment for coronary artery perforation (CAP), but without satisfied late-term outcomes when compared to drug-eluting stents (DES). This study seeks to report a new covered stent to treat porcine CAP, which is manufactured with DES and a biodegradable membrane fabricated by poly-L-lactic acid (PLLA) polymer.

**Methods:**

Experimental swines experienced CAP in proximal-middle of right coronary artery (RCA) by non-compliant balloon burst, and covered stent was deployed in breach segment. Meanwhile, coronary angiography (CAG), optical coherence tomography (OCT), histological light microscopy and scan electron microscopy were performed to characterize the performance of covered stent.

**Results:**

Seven swines were used for this study. Two swines were euthanasia at 14 days and 28 days after procedure, respectively. The remaining 5 kept alive until sacrifice at six months. CAG at six months showed total occlusion at the stented segment of RCA in all swines. The interventional revascularization of occlusion lesion was instituted in two swines. After recanalizing occlusion lesion, OCT examination visualized diffuse heterogeneous fibrous plaques, as well as organized thrombosis, lipid deposits and several neoatherosclerosis in the occluded segment. Serial histopathologic and electron microscopies at 14 days, 28 days and six months revealed gradual occlusive vessel lumen with diffuse heterogeneous fibroplasia, smooth muscle proliferation, inflammation response and local neoatherosclerosis, moreover with identification of PLLA polymer membrane degradability.

**Conclusions:**

The new covered stent with biodegradable membrane could seal urgent coronary breach and prevent experimental swines death, but with all stent occlusion in mid-term (six months) follow-up, which might be attributed to diffuse heterogeneous fibroplasia, smooth muscle proliferation, inflammation response and local neoatherosclerosis with the degradation of PLLA membrane.

## 1. Introduction

Coronary artery perforation (CAP) is a rare but life-threatening complication of percutaneous coronary intervention (PCI), with 0.1%–2.5% incidence and greater than 20% mortality [1, 2]. Except surgical drainage, various interventional approaches (such as embolization with coils or autologous fat particles, prolonged balloon inflation and covered stent) have been applied to treat CAP [2, 3, 4]. Among these, covered stent is the only one that provides the physical barrier to seal emergency coronary breach while maintaining the antegrade coronary artery flaw, when prolonged balloon dilation was ineffective [5].

The first historic coronary covered stents were made with autologous veins or arterial walls, but without any benefit clinical outcomes when compared to bare metal stents [6]. Currently, covered stents have rapidly evolved and widely applied in clinical practice. They are divided into three commercially available covered stents: polytetrafluoroethylene (PTFE) (Direct-Stent, BeGraft coronary stent graft system, Graftmaster), polyurethane (PK Papyrus stent) and pericardium covered stent (second generation pericardial stents Aneugraft Dx stent, “Over and Under OU”—first-generation pericardial stents) [7]. Besides, self-made polyurethane covered stent had been also reported when lack of commercial covered stent [3]. Previous studies have demonstrated that covered stents could reduce the risk of morbidity and mortality [7, 8, 9, 10, 11], however all clinically available covered stents were unable to achieve satisfied late-term outcomes when compared to drug-eluting stents [5, 12, 13].

Hence, we seek to report a new self-made covered stent to treat porcine CAP, which is manufactured with a second generation drug-eluting stent and an expandable membrane fabricated by biodegradable poly-L-lactic acid (PLLA) polymer. Meanwhile coronary angiography (CAG), optical coherence tomography (OCT), histological light microscopy (HLM) and scan electron microscopy (SEM) have also been applied to characterize mid-term (six months) performance of the new covered stent.

## 2. Materials and Methods

### 2.1 Characteristics of Self-Made Covered Stent 

**Fig. 1.**
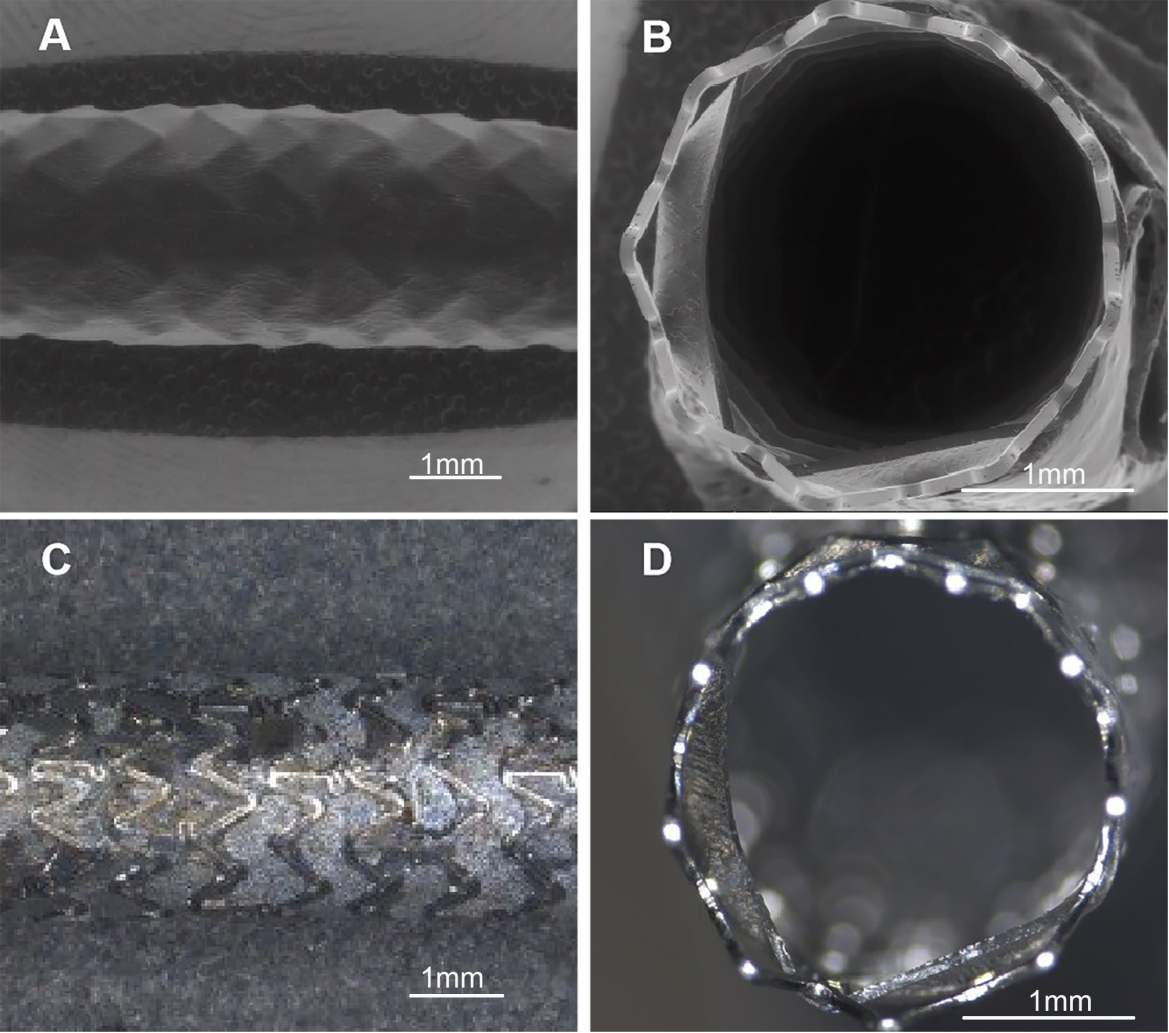
**Microphotographs and digital photographs of covered stent**. (A) Microphotograph of overall covered stent (10×). (B) Microphotograph of cross-sectional covered stent (20×). (C) Digital photograph of overall covered stent (10×). (D) Digital photograph of cross-sectional covered stent (20×).

The self-made covered stent is composed of a second generation drug-eluting stent (DES) (Firebird 2, MicroPort Medical Co., Shanghai, China) and an expandable membrane fabricated by biodegradable PLLA polymer. The degradable membrane can be reduced to 20%–40% in 3–6 months when coated in a 37 °C buffer solution, as well as the highly expandable biodegradable PLLA polymer, with a thickness of 60–80 µm before dilatation, and 20–40 µm after complete dilatation [2]. The specification of the single layer covered stent is 2.5 mm × 29 mm, shown in Fig. 1.

### 2.2 Seal Porcine Coronary Breach

Juvenile Yorkshire swines (15–20 kg) were given loading doses of aspirin (300 mg) and clopidogrel (300 mg) 24 hours prior to catheterization and maintained with aspirin (100 mg daily) and clopidogrel (75 mg daily) until sacrifice. All experimental procedures were performed in accordance with the National Institutes of Health guidelines for humane handling of animals and were approved by the Institutional Animal Care and Use Committee of Fujian Medical University (FJMU IACUC 2019-0070).

The procedure in Cath Lab was done under induction of anesthesia with an intramuscular injection of a mixture of tiletamine and zolazepam (2.5 mg/kg, Zoletil50, Virbac, Carros, France), and maintenance of anesthesia with inhaled sevoflurane and analgesia with fentanyl. Meanwhile, mechanical ventilation was performed in all swines. A 6 Fr vascular sheath (Terumo Co., Tokyo, Japan) was placed in the right femoral artery. After infusion of 100 IU/kg heparin, a 6 Fr Judkins Right 4.0 guiding catheter (Cordis Co., Santa Clara, CA, USA) was engaged in the right coronary artery (RCA) under fluoroscopic guidance. One 0.035-inch Runthrough guidewire (Terumo Co., Tokyo, Japan) was sent to the distal of RCA, and non-compliant balloon Quantum (Boston Scientific, Marlborough, MA, USA) 3.25 × 12 mm was successively placed in the proximal-middle of RCA and dilated beyond the burst pressure of the balloon to create coronary perforation.

**Fig. 2.**
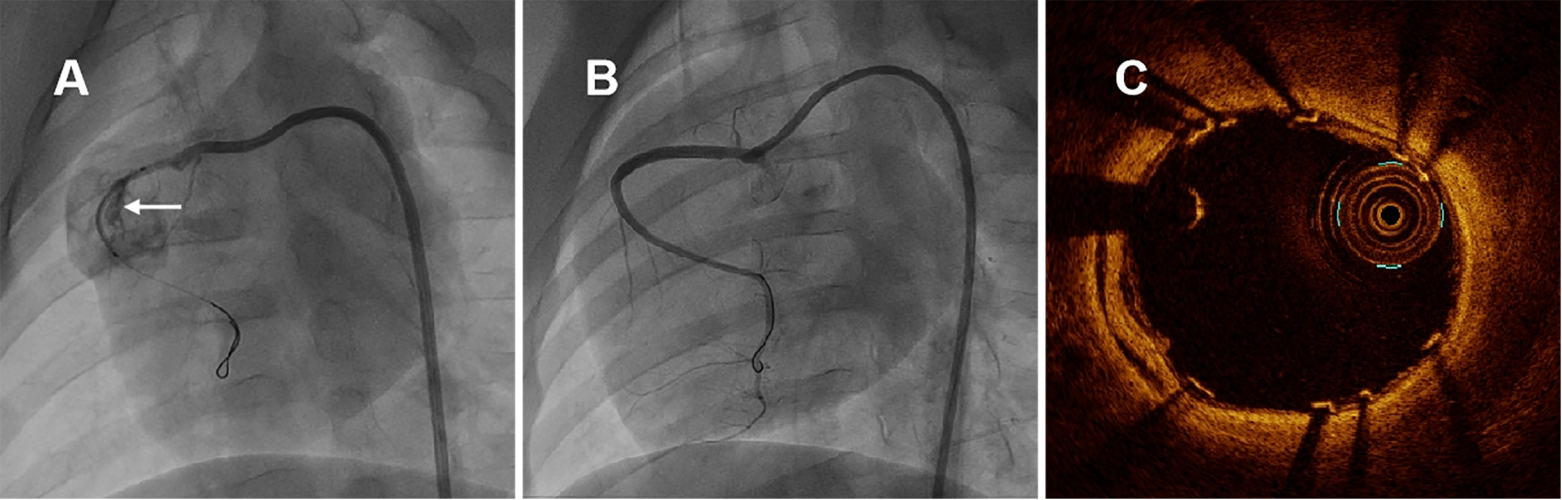
**Representative coronary angiographies and OCT image in swine immediately after covered stenting**. (A) Coronary artery perforation was induced by non-compliant balloon burst and self-made covered stent was positioned in RCA, the white arrow indicated the perforation of coronary nature architecture. (B) CAG of RCA immediately after covered stenting. (C) OCT examination immediately after covered stenting. CAG and OCT examination revealed that the new covered stent was sufficient expansion without malapposition, meanwhile with pretty blood flaw. OCT, optical coherence tomography; RCA, right coronary artery; CAG, coronary angiography.

Subsequently, self-made covered stent was deployed to seal the breach of RCA, shown in Fig. 2A. Finally, CAG and OCT examinations were performed immediately after stent implantation. When ventricular arrhythmia happened, electrical defibrillation, cardiopulmonary resuscitation and drug therapy were carried out. The swines were allowed to recover and resume feeding for subsequent study.

### 2.3 Follow Up

Surgical dissection was performed to expose the right femoral artery under anesthesia, analgesia and mechanical ventilation at six months after stent implantation. A 6 Fr vascular sheath was placed in the right femoral artery. Angiography of RCA and left coronary artery (LCA) were instituted by 6 Fr Judkins Right 4.0 guiding catheter (Cordis Co., Santa Clara, CA, USA). After the procedure, the swines were euthanasia and the stented coronary arteries were sectioned and fixed by immersion in a buffered formalin solution and glutaraldehyde solution respectively, to perform HLM and SEM examinations by experienced specialists.

## 3. Results

A total of seven juvenile Yorkshire swines were used for this study. One swine suffered from ventricular fibrillation during the OCT examination immediately after stent implantation and survived by electrical defibrillation. Two swines were euthanasia at 14 days and 28 days after procedure, respectively. The remaining 5 were sacrificed at six months. During coronary artery injury in RCA induced by balloon burst, no hemodynamic disorder had been detected in these animals during the procedure. Angiographic and OCT examinations immediately after stent implantation showed that the self-made covered stent had completely sealed the breach segment, achieving good apposition of the stent to vessel wall, as well as pretty antegrade blood flaw-thrombolysis in myocardial infarction (TIMI) grade 3, shown in Fig. 2B,C.

**Fig. 3.**
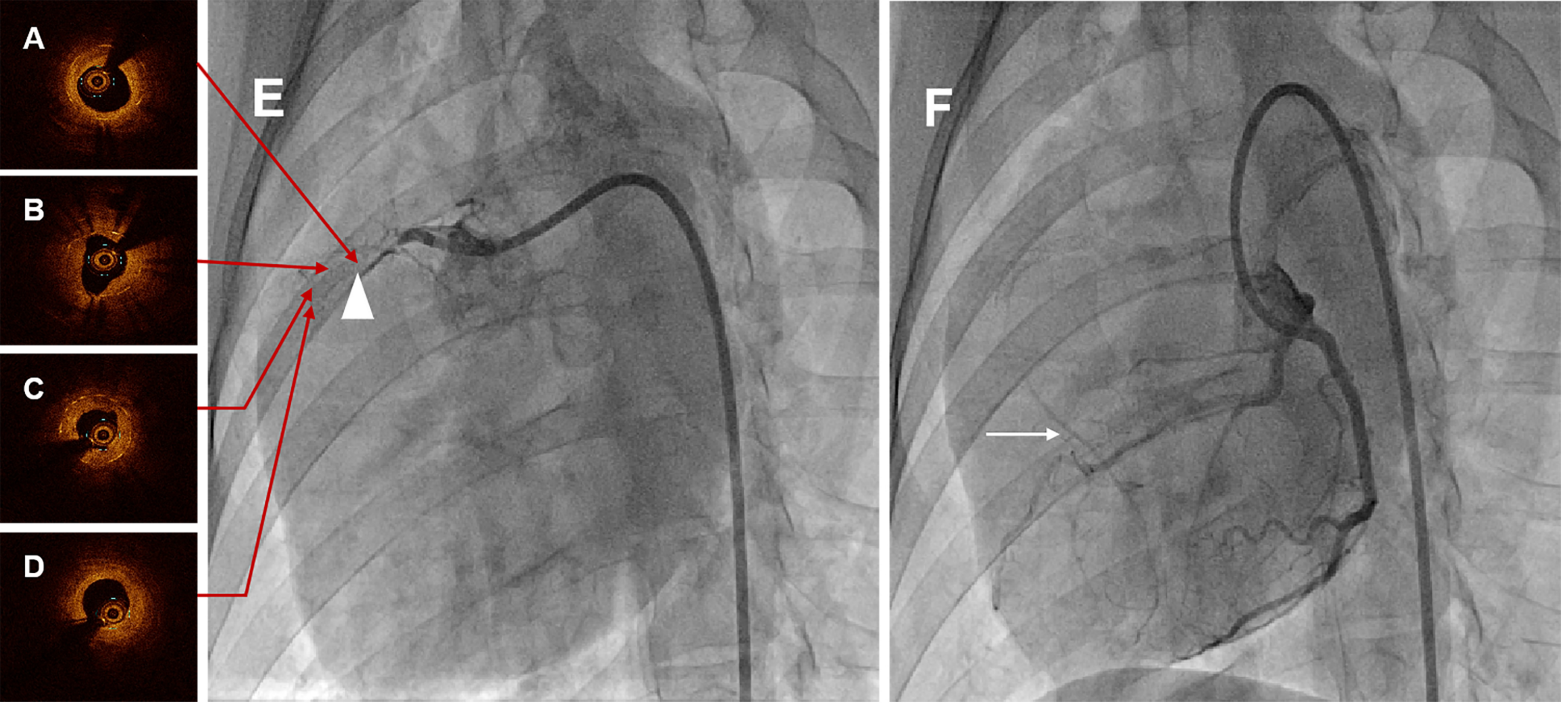
**Representative coronary angiographies and OCT images in swine at six months**. (A) Proximal OCT image of the occlusion lesion after interventional recanalization. (B) Proximal-middle OCT image of the occlusion lesion after interventional recanalization. (C) Middle-distal OCT image of the occlusion lesion after interventional recanalization. (D) Distal OCT image of the occlusion lesion after interventional recanalization. Serial OCT images visualized the diffuse heterogeneous fibrous plaques, as well as organized thrombosis, lipid deposits and several neoatherosclerosis in the occluded segment. (E) CAG of RCA in swine at six months, and the white triangle indicated the occluded stented lesion. (F) CAG of LCA in swine at six months, and the white arrow was the collateral circulation. CAG at six months showed total occlusion at the stented segment with pretty collateral circulation. OCT, optical coherence tomography; CAG, coronary angiography; RCA, right coronary artery; LCA, left coronary artery.

CAG at six months demonstrated total occlusion at the proximal-middle stented RCA segment in all swines, meanwhile with pretty collateral circulation (Rentrop grade 3), shown in Fig. 3E,F and **Supplementary Movie 1 **and **Supplementary Movie 2**. Among these animals, two swines were used to constitute occluded stented lesion interventional revascularization and perform the OCT examination. OCT images visualized the diffuse heterogeneous fibrous plaques, as well as organized thrombosis, lipid deposits and several neoatherosclerosis in the occluded segment, shown in Fig. 3A–D and **Supplementary Movie 3**.

**Fig. 4.**
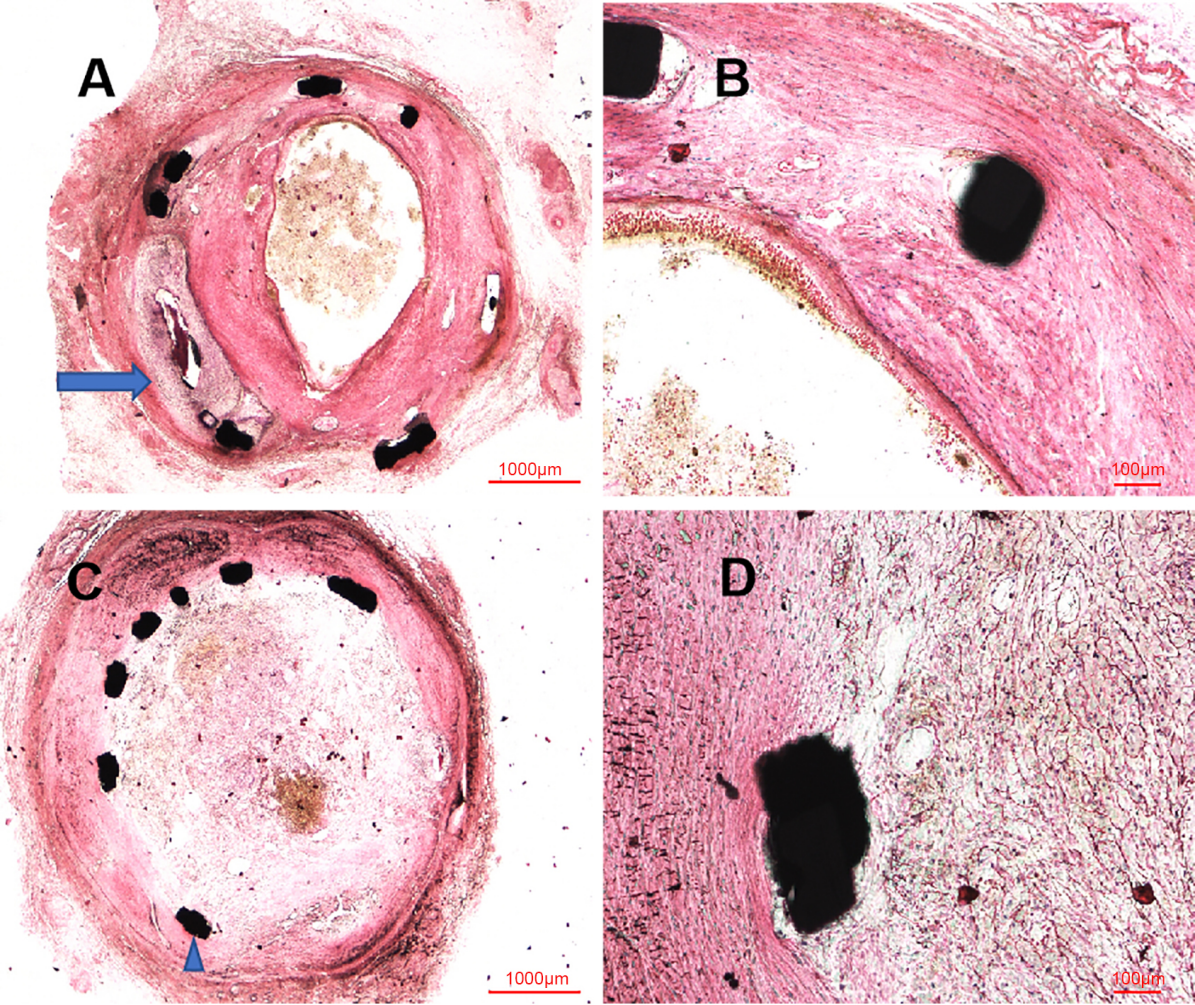
**Representative HLM images by toluidine blue-fuchsin stain in swines with and without interventional recanalization at six months**. (A) Histological image in swine with interventional recanalization (2×), and the blue arrow was the site of coronary artery perforation. (B) Histological image in swine with interventional recanalization (10×). (C) Histological image in swine without interventional recanalization (2×), and the blue triangle was the struct of covered stent. (D) Histological image in swine without interventional recanalization (10×). These HLM images showed organized thrombosis with small plaques nourish vessels, blood clots and blood cells filling the vessel lumens, in the context of fibroplasia, smooth muscle proliferation and inflammatory cells near the stent structs. HLM, histological light microscopy.

**Fig. 5.**
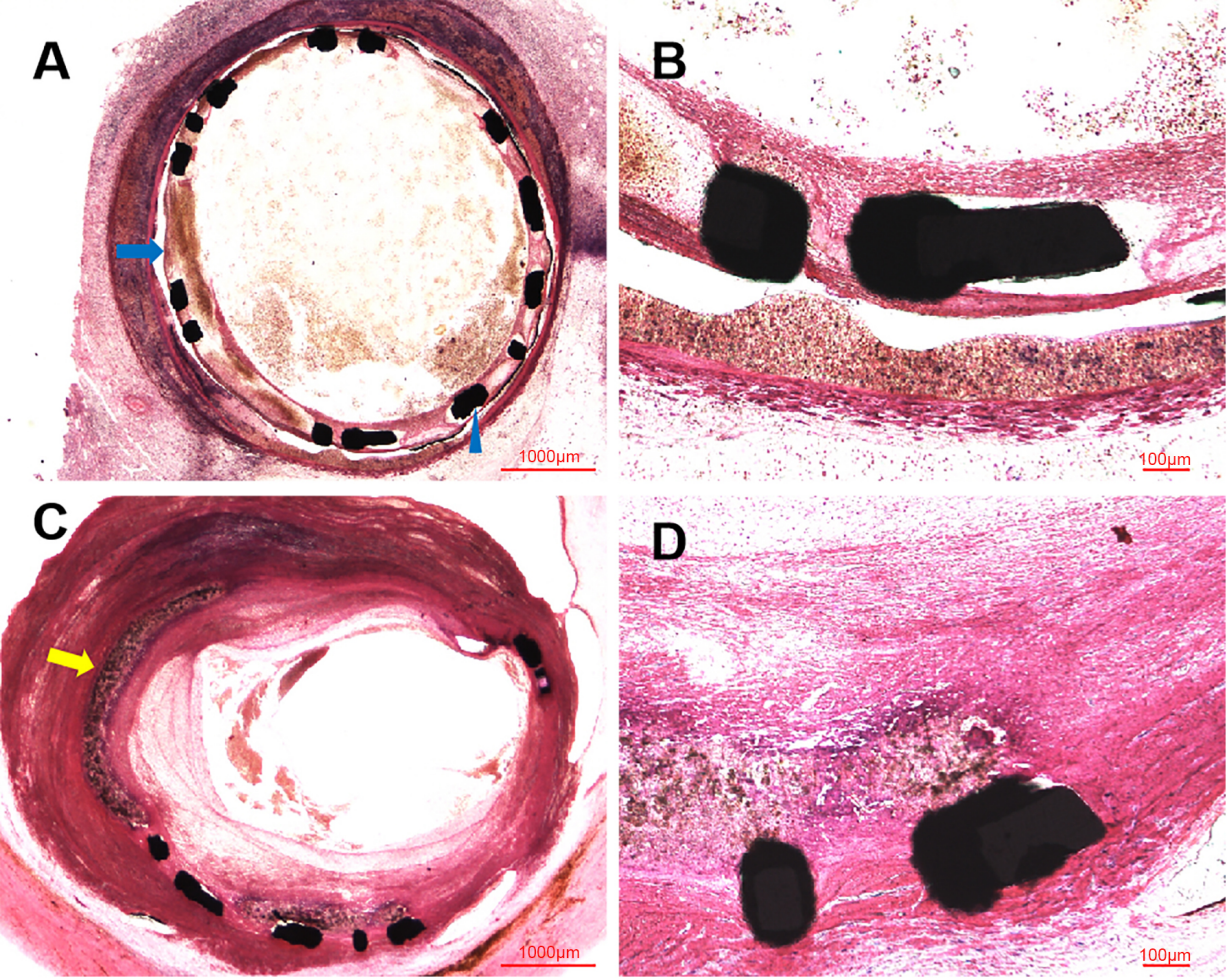
**Representative HLM images by toluidine blue-fuchsin stain in swines at 14 and 28 days**. (A) Histological image in swine at 14 days (2×), and the blue arrow was the site of biodegradable PLLA polymer membrane, the blue triangle was the struct of covered stent. (B) Histological image in swine at 14 days (10×). Histological images at 14 days revealed the patent vessel lumen with initial endothelization process of self-made covered stent and inflammatory cells along the identified PLLA polymer membrane. (C) Histological image in swine at 28 days (2×), and the yellow arrow was the site of coronary artery perforation. (D) Histological image in swine at 28 days (10×). Histological images at 28 days showed the patent vessel lumen with fibroplasia, endothelial proliferation and inflammation response. HLM, histological light microscopy; PLLA, poly-L-lactic acid.

Similar to the OCT examination, histopathologic examination at six months showed the organized thrombosis with small plaques nourish vessels, blood clots and blood cells filling the vessel lumens, in the context of fibroplasia, smooth muscle proliferation and inflammatory cells near the stent structs, shown in Fig. 4. Moreover, the site of CAP induced by balloon burst was visualized in these histopathologic images. Nevertheless, the vessel lumens had been kept patent in the early time after procedure, shown in Fig. 5, with initial endothelization process of self-made covered stent and inflammatory cells along the identified PLLA membrane at 14 days (Fig. 5A,B), as well as fibroplasia, smooth muscle proliferation and inflammation response at 28 days (Fig. 5C,D).

**Fig. 6.**
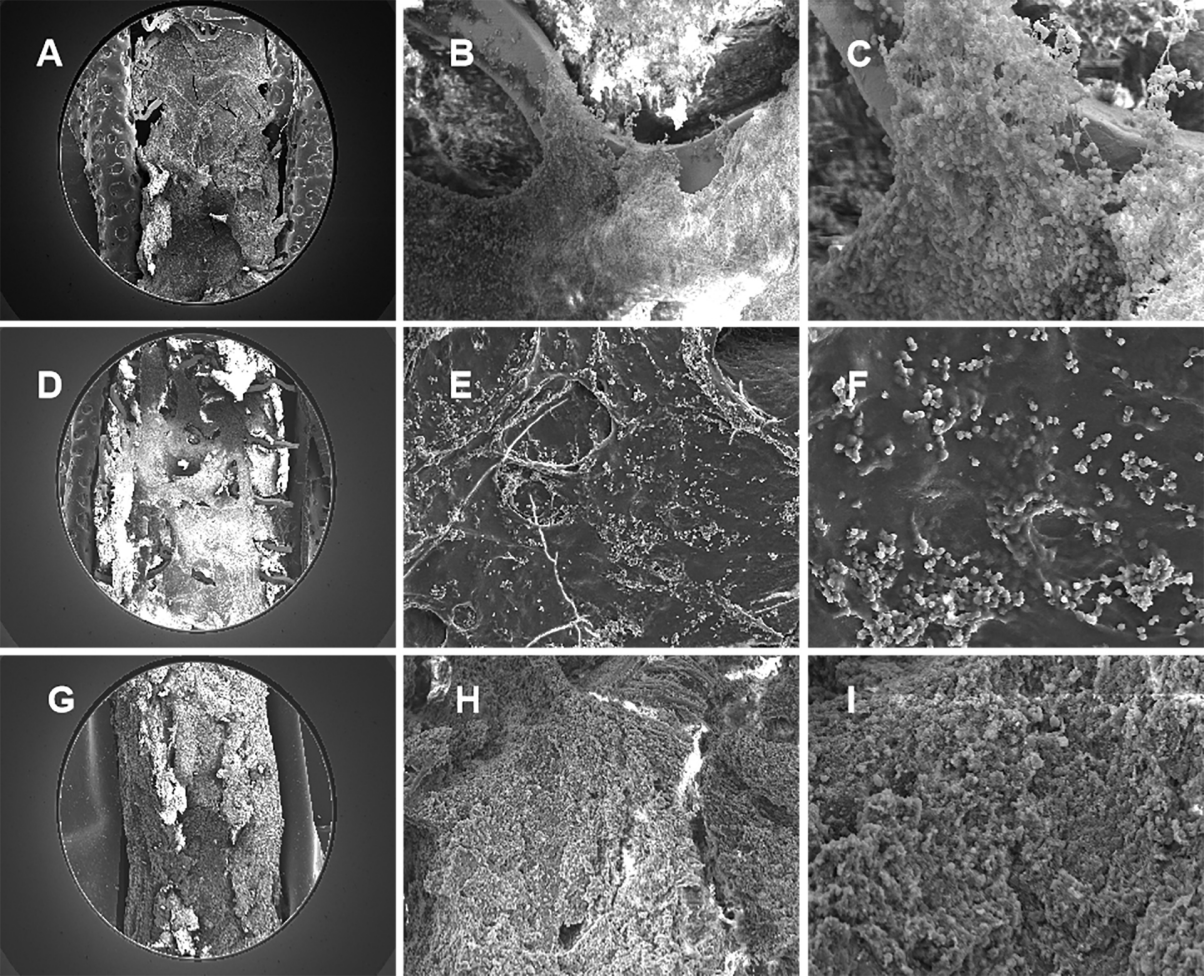
**Representative SEM images in swines at 14, 28 days and six months**. (A) SEM image in swine at 14 days (100×). (B) SEM image in swine at 14 days (300×). (C) SEM image in swine at 14 days (1000×). The electron-microscopies at 14 days showed endothelial cells inhomogeneously crawling on the scaffold structs. (D) SEM image in swine at 28 days (100×). (E) SEM image in swine at 28 days (300×). (F) SEM image in swine at 28 days (1000×). The electron-microscopies at 28 days revealed tight cell contact in the scaffold segment. (G) SEM image in swine at six months (100×). (H) SEM image in swine at six months (300×). (I) SEM image in swine at six months (1000×). The electron-microscopies at six months showed fibrotic tissue and blood cells filled to full the vessel lumen. SEM, scan electron microscopy.

The serial SEM images was shown in Fig. 6, with gradual occlusion in RCA stented segment. Compared with the SEM images at 28 days shown in Fig. 6D–F, which was actively proliferation, with tight cell contact in the scaffold segment, the electron-microscopy at 14 days showed the endothelial cells inhomogeneously crawling on the scaffold structs, shown in Fig. 6A–C. Nevertheless, at six months, the fibrotic tissue and blood cells filled to full the vessel, shown in Fig. 6G–I, which rather differed from the electron-microscopies at 14 days and 28 days.

## 4. Discussion

This study is an investigation of new covered stent evolution, focusing on biodegradable PLLA membrane. In our hypothesis, DES covered with biodegradable PLLA membrane could gradually degrade with the repair of CAP, finally without membrane in coronary artery, to achieve equivalent clinical outcomes with drug-eluting stent. In order to further evaluate the safety and efficiency of new covered stent with biodegradable membrane, we performed this study and found that the new covered stent with biodegradable membrane could seal urgent coronary breach and prevent experimental swines death, but with all stent occlusion in mid-term (six months) follow-up.

To our knowledge, the incidence of CAP has not decreased with the development of PCI techniques and devices, oppositely with an increased tendency for the complex lesions interventional revascularizations [14, 15, 16]. In order to effectively and timely treat urgent CAP, device deliverability had to play an important role in procedure, especially in tortuous and calcified vessels. Historic autologous vein/artery covered stents had been abandoned for the lower fixation deliverability and cumbersome preparation process [17]. The mainstream commercial covered stents are 1.63–1.73 mm crossing profile PTFE-coated stent with two-sent layer design (Direct-Stent, Graftmaster), 105 µm equine pericardium stent (Aneugraft), 90 µm single layer PTFE-coated stent (BeGraft), and 60 µm polyurethane-coated stent (PK Papyrus) [12]. Benefiting from the smaller crossing profile, new generation single layer covered stents (BeGraft and PK Papyrus) had better performance on flexibility and deliverability [12]. As reported by Kufner *et al*. [18], BeGraft covered stent had been demonstrated highly deliverable, with 96.7% technique success rate in 61 CAP patients. Additionally, lower device delivery time (8 *vs.* 15 min, *p *= 0.001) was indicated in 22 CAP patients treated with PK Papyrus, when compared to 39 CAP patients treated with Jostent Graftmaster, despite with greater stent length in PK Papyrus group (20 ± 5 *vs*. 16 ± 3 mm,* p* < 0.001) [11]. Kandzari *et al*. [16] had also reported successful delivery in 95% of cases (total CAP n = 80) in patients treated by PK Papyrus, which was consistent with the results of the French multicenter study and the Papyrus Spain Registry, where the delivery success rates in 130 and 52 CAP patients treated by PK Papyrus were 95%, 94%, respectively [19, 20].

Even so, the procedural death in CAP patients was still undesirable, with a range of 8.2%–14.8%, originating from the inherent risks of perforations [5, 9, 16, 19, 21]. The new generation of single layer PTFE covered stent (BeGraft) had also been reported with similar outcomes, with 8.2% cardiac death in hospital [18, 22]. Of note, all swines in our study had been successfully implanted with new covered stents, and kept alive during the interventional procedure, without cardiac tamponade. The immediate postoperative imaging evaluation had also indicated the novel covered stent could completely seal coronary breach, meanwhile with good apposition of the stent to vessel wall and pretty antegrade blood flow (TIMI grade 3). As related to the safety and efficiency in the short-term, the performance of our new covered stent seemed satisfied.

However, the ending of the novel covered stent was depressing, especially in in-stent restenosis (ISR). It was several comforting that all experimental swine kept alive at six months follow-up, except two swine were euthanasia at 14 days and 28 days to get the early evaluation of HLM and SEM. Nagaraja *et al*. [23] reported current covered stents applied for the treatment of CAP were associated with high long-term mortality, with 18.5% in Craftmaster, 16.0% in PK Papyrus and 26.1% in pericardial stents respectively, which seemed to be higher than BeGraft covered stent (11.5% in 61 CAP patients) [18]. The long-term mortality in self-made polyurethane covered stent was consistent with commercial covered stents, with a report of 22.7% cardiac death [3].

In a previous observational study, Rosseel *et al*. [24] had reported a high rate of ISR (29.2%) in twenty-four patients treated with double layer PTFE-coated and single layer pericardium covered stents. This was comparable with other studies, with 31.6% up to 54.6% ISR rate in patients treated with sandwich PTFE-coated covered stent [10, 25], and 26.3% in pericardium covered stent [26]. The new generation single layer PTFE-coated covered stent had also been reported 18% long-term incidence of target lesion revascularization (TLR) and without any cases of stent thrombosis in only 42.6% (n = 61) available angiographic follow-up [18]. As for PK Papyrus covered stent, the late-term outcomes seemed to be inconsistent when compared to PTFE-coated covered stent [23, 27]. TLR in long-term follow-up were reported in prior studies, with a 16% TLR and 8% definite stent thrombosis in the SOS PK Papyrus Registry (n = 127), a 3.8% TLR with no stent thrombosis in the Spain Papyrus Registry (n = 52) and a 9.3% TLR and 4.4% stent thrombosis in the Swedish SCAAR Registry (n = 265) [13, 19, 20].

In our study, all experimental swines had failed in the performance of ISR. The CAG at six months follow-up showed that the proximal-middle stented RCA segment with total occlusion with pretty collateral circulation. In an effort to explore the underlying pathophysiological components of total occlusion, we had performed total occlusion lesion interventional revascularization and subsequent OCT examination, which had indicated the total occlusion lesions were filled with diffuse heterogeneous fibrous plaques, organized thrombosis, lipid deposits and several neoatherosclerosis. A series of chronological histopathologic examination in our study had also revealed the gradual occlusive vessel lumen with fibroplasia, smooth muscle proliferation and inflammation response near the degradable membrane, meanwhile with the identification of PLLA polymer membrane degradability.

The underlying pathogenesis of ISR in our new covered stent was inconsistent with other commercial covered stent. The construction of commercial covered stents with nondegradable membrane predisposed to increase inhomogeneous neointimal hyperplasia in the edge location and time course [23, 27]. In the case reported by Araki *et al*. [28], a saphenous vein graft perforation patient was treated by PTFE-coated covered stent, and suffered from ISR in the distal edge of covered stent at 9-months follow-up. OCT and coronary angioscopy images indicated the neointimal characteristics of restenosis, with delayed endothelization. Not alone this, previous reports also described the relation between ISR and inhomogeneous delayed neointimal hyperplasia [10, 29, 30, 31]. Nevertheless, the ISR of swines in our study would be attributed to diffuse heterogeneous fibroplasia, smooth muscle proliferation, inflammation response and local neoatherosclerosis with the degradation of PLLA membrane. As indicated by prior studies about biodegradable polymer sirolimus-eluting stent [32, 33], the vascular responses for biodegradable polymer absorption could potentially intensify local inflammation, which might lead to delay arterial healing, accelerate fibrocytes and smooth muscle cells proliferation, all leading to cause ISR. Remarkably, the ISR of our new novel covered stent was different from current DES. Nakazawa *et al*. [34] retrospectively analyzed 299 consecutive autopsy cases with 406 coronary lesions (197 treated with bare mental stent and 209 treated with DES), and found that neoatherosclerosis in DES group occurred earlier than in bare mental stent group, due to the delayed healing and endothelialization in DES group would accelerate infiltration of lipids [35]. From our clearer and more visualized serial OCT images, histopathologic images and electron microscopies, we hypothesized that the degradation of PLLA membrane had strengthen local inflammation response and delayed the perforation healing, simultaneously promoting fibrocytes and smooth muscle cells excessive proliferation. During the process of gradual occlusion, local thrombosis and neoatherosclerosis had also played positive roles.

The underlying processes responsible for the development of ISR following our covered stent implantation were likely multifactorial. In our previous study, we had confirmed the long-term efficacy and safety of the biodegradable PLLA membrane in rabbit abdominal aorta bifurcation [12], however the late-term performance of the biodegradable PLLA membrane was disappointed when encountering the large laboratory porcine and adjunctive CAP. For consideration, the large laboratory swine may fail to control the complete intake of antiplatelet drugs [36]. Besides that, the CAP induced by balloon burst was uncertain and inconsistent in the depth and length of segment coronary artery injured. Although the new covered stent could successfully seal the coronary breach, the hematoma and dissection occurring in distal coronary artery were probably unable to be completely sealed.

Our study possesses several limitations. Firstly, we did not evaluate the detail of covered stent gradual occlusion process at various time points shorter than six months, as well as the undetermined degradation curve of PLLA polymer *in vivo*. Secondly, no histologic differences were observed between the proximal and distal caps. Fortunately, OCT examination could reflect these histological changes to some degree. Another was the limited animal sample in our study. Further researches are necessary to develop and improve coronary covered stent.

## 5. Conclusions

This new covered stent with biodegradable membrane could seal urgent coronary breach and prevent experimental swines death, but with all stent occlusion in mid-term (six months) follow-up, which might be attributed to diffuse heterogeneous fibroplasia, smooth muscle proliferation, inflammation response and local neoatherosclerosis with the degradation of PLLA membrane. Further researches are necessary to develop and improve coronary covered stent.

## Data Availability

The datasets used and analyzed during the current study are available from the corresponding author on reasonable request.

## Abbreviations

CAP, coronary artery perforation; PTFE, polytetrafluoroethylene; PLLA, poly-L-lactic acid; CAG, coronary angiography; OCT, optical coherence tomography; HLM, histological light microscopy; SEM, scan electron microscopy; RCA, right coronary artery; ISR, in-stent restenosis.

## Supplementary Material

Supplementary material associated with this article can be found, in the online version, at https://doi.org/10.31083/j.rcm2407197.
